# Methods for Sterilizing Clinically Relevant Wear Particles Isolated from Metal-on-Metal Hip Implants

**DOI:** 10.1038/s41598-017-18239-4

**Published:** 2018-02-05

**Authors:** Ernest S. Fung, Kenneth M. Unice, Dennis J. Paustenbach, Brent L. Finley, Michael Kovochich

**Affiliations:** 1 Cardno ChemRisk, 65 Enterprise Suite 150, Aliso Viejo, CA 92656 USA; 2 Cardno ChemRisk, 20 Stanwix St. Suite 505, Pittsburgh, PA 15222 USA; 3 Cardno ChemRisk, 101 2nd St. Suite 700, San Francisco, CA 94105 USA; 4 Cardno ChemRisk, 231 Front Street Suite 212, Brooklyn, NY 11201 USA; 5 Cardno ChemRisk, 30 North LaSalle St Suite 3910, Chicago, IL Illinois 60602 USA

## Abstract

Engineered or incidental particles may contain endotoxin from contaminated environments associated with generation, production, or handling activities. Endotoxins are ubiquitous contaminants that may yield false positive responses in immunological assays if present. The purpose of this study was to develop a sterilization method for removal of endotoxin from clinically relevant wear particles isolated from metal-on-metal (MoM) hip implant lubricant. In this case, the goal of particle sterilization was to sufficiently reduce endotoxin levels to acceptable levels for sensitive biological assays while retaining the physical and chemical characteristics of the original particles. Optimization of treatment with 0.05 NaOH in 50% ethanol successfully achieved a 5-log (>99.999%) reduction of endotoxin content while retaining the size and chemistry of MoM hip implant wear particles. Using the optimized method, the concentration of endotoxin was reduced from 161,000 to 1.19 EU/mL. As particle types can vary, sterilization strategies will also differ to optimize endotoxin removal while retaining key particle characteristics. To our knowledge, this study represents the first published sterilization method for clinically relevant MoM hip implant wear particles isolated from serum-rich lubricant.

## Introduction

There is an extensive amount of ongoing research focused on the potential contribution of metal particles and ions released from metal-on-metal (MoM) hip implants to observed biological responses^1,2^. However, many previous toxicological studies have utilized particles with physical and chemical characteristics that differ from particles released from MoM implants under normal wear conditions^1,2^. For example, numerous studies have evaluated the biological responses (*in vitro* and *in vivo*) induced by metal particles collected from hip simulators that were operated in an aqueous solution with no protein lubricant^3–6^. The main limitation of this study design is that the wear particles that are generated in water display significant chemical differences (more cobalt) compared to particles that are generated under physiologically relevant conditions with serum-containing lubricant solution^1,2^.

Our goal, therefore, was to collect wear particles from MoM hip simulators operated in a physiologically relevant serum-rich lubricant for potential toxicological testing. Some of the challenges with particles collected in a serum-rich lubricant include the need to digest proteins prior to any testing along with potential endotoxin contamination that can be common during various handling activities. Recently, we successfully characterized the physical and chemical characteristics of wear particles released from MoM hip implants under normal versus edge loading conditions^7^. In order to test the potential biological responses associated with these clinically relevant particles, it was critical to properly sterilize and remove any potential endotoxin contamination.

Endotoxin or lipopolysaccharide (LPS) is a large (up to 1000 kDa), chemically diverse, heat stable component found on the outer membrane of Gram-negative bacteria^8,9^. Endotoxin is a ubiquitous contaminant found in many different environments including the air, drinking water, dust, and surfaces^10–12^. Moreover, contamination is common in laboratories, during biomaterial and pharmaceutical production, and on surfaces such as particles^9,10,13–19^. Endotoxin contamination of test articles can present a challenge when investigating immune responses in biological systems due to “false positive” results that are unrelated to the material being evaluated^14,18–20^. The goal of “depyrogenation” is to reduce endotoxin content to levels that do not elicit an inflammatory response.

The purpose of this communication, therefore, is to describe an optimized sterilization method for effective endotoxin removal from clinically relevant MoM wear particles isolated from serum-rich lubricant. To our knowledge, there are no published methods for sterilizing clinically relevant wear particles from serum-rich lubricant. The method described in this paper yields sterilized particles that retained the physical and chemical properties of clinically relevant wear particles. Specifically, our method resulted in a significant reduction of endotoxin to levels that are unlikely to induce a positive response in sensitive immunological assays while retaining the chemical content (% of particle mass comprised of Co and Cr) and particle morphology (size and shape). The development of a reliable sterilization technique will allow further study of clinically relevant laboratory simulated implant wear particles in various *in vitro* and *in vivo* systems.

## Results

**Figure 1:**
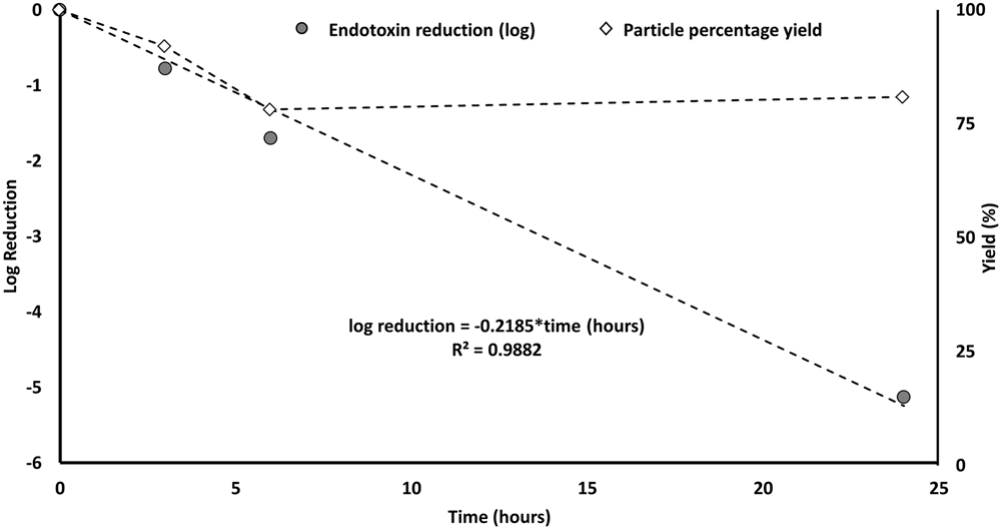
Endotoxin reduction and particle yield as a function of time. Log reduction of endotoxin content and percentage yield (total mass) in MoM wear particles was determied over time after treatment with 0.05 M NaOH.

The endotoxin content of wear debris particles prior to sterilization was 161,000 EU/mL. Prior to sterilization treatment, the wear debris particles were comprised of 1.35% Co and 98.65% Cr (Table 1), had a mean primary particle size of 55 nm by number, and were primarily round and oval in shape (Table 2). Initial testing showed that 0.05 M sodium hydroxide treatment efficiently reduced the endotoxin content without altering the physical and chemical characteristics of the wear debris particles. Thus, we proceeded to optimize the sodium hydroxide treatment for increased endotoxin removal efficacy. Specifically, we evaluated the percentage of endotoxin removed over the course of 3, 6, and 24 hours of NaOH treatment, and found that the endotoxin level was reduced by 83.354, 98.012, and 99.999% after 3, 6, and 24 hour of 0.05 M NaOH treatment, respectively (Fig. 1; Table 1). Absolute endotoxin content was reduced from 161,000 to 26,800, 3,200, and 1.19 EU/mL after 3, 6, and 24 hour of 0.05 M NaOH treatment (Table 1). We then determined percent particle yield (total mass) as well as chemical characteristics of the treated wear particles under these same conditions.

**Table 1:** Optimization of NaOH sterilization method.

Treatment	Endotoxin content (EU/mL)^1^	% Endotoxin Reduction	% Particle Yield	% Cobalt^2^	% Chromium^2^
Before treatment	161000	—	—	1.35	98.65
0.05 M NaOH (3 hrs)	26800	83.354	92.16	1.64	98.36
0.05 NaOH (6 hrs)	3200	98.012	78.15	1.50	98.50
0.05 NaOH (24 hrs)	1.19	99.999	80.75	1.50	98.50

^1^Endotoxin content determined by LAL assay; ^2^Analyzed by ICP-MS.

**Table 2:** Primary particle size and morphology of 0.05 M NaOH-treated wear particles.

	Diameter (nm)						Morphology (%)		
	Average	Median	Mode	St Dev	Min	Max	Round	Oval	Rod
**Before treatment**									
By number	54.78	44.33	30.11	42.40	10.63	417.60	62.5	35.0	2.5
By volume	89.90	44.33	30.11	160.17	10.63	417.60			
**0**.**05** **M NaOH treatment for 24** **hours**									
By number	46.16	35.97	19.74	38.68	11.36	414.69	60.0	36.4	3.6
By volume	81.49	35.97	19.74	156.17	11.36	414.69

Particle yield was determined to be 92, 78, and 81% after 3, 6, and 24 hour treatment of 0.05 M NaOH treatment, respectively (Fig. 1; Table 1). ICP-MS was used to determine the chemical composition of particles before and after 0.05 M NaOH treatment (Table 1). Prior to 0.05 M NaOH treatment, the particles were comprised of 1.35% Co and 98.653% Cr. The Co content of the particles was 1.64, 1.50, and 1.50% after 3, 6, and 24 hour 0.05 M NaOH treatment, respectively. The Cr content of the particles was 98.36, 98.50, and 98.50% after 3, 6, and 24 hour 0.05 M NaOH treatment. Hence, NaOH treatment had no effect on the chemical composition of the particles, regardless of treatment duration.

**Figure 2:**
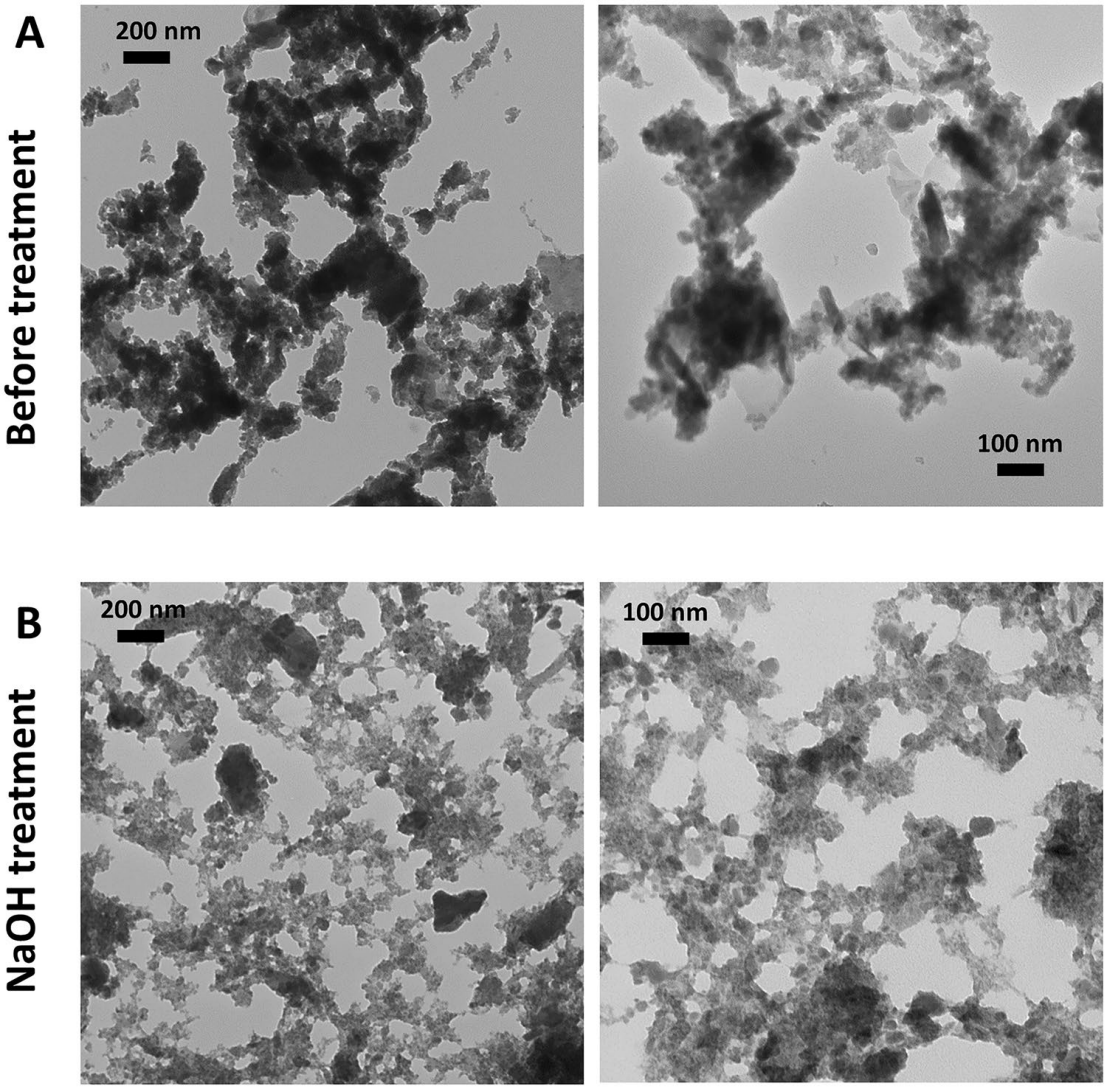
0.05 M NaOH-treatment does not alter physical characteristics of MoM Wear Particles. TEM analysis was performed to assess MoM wear particles: (**A**) prior to treatment at 10X and 20X magnification; (**B**) after 0.05 M NaOH treatment for 24 hours at 10X and 20X magnification.

**Figure 3:**
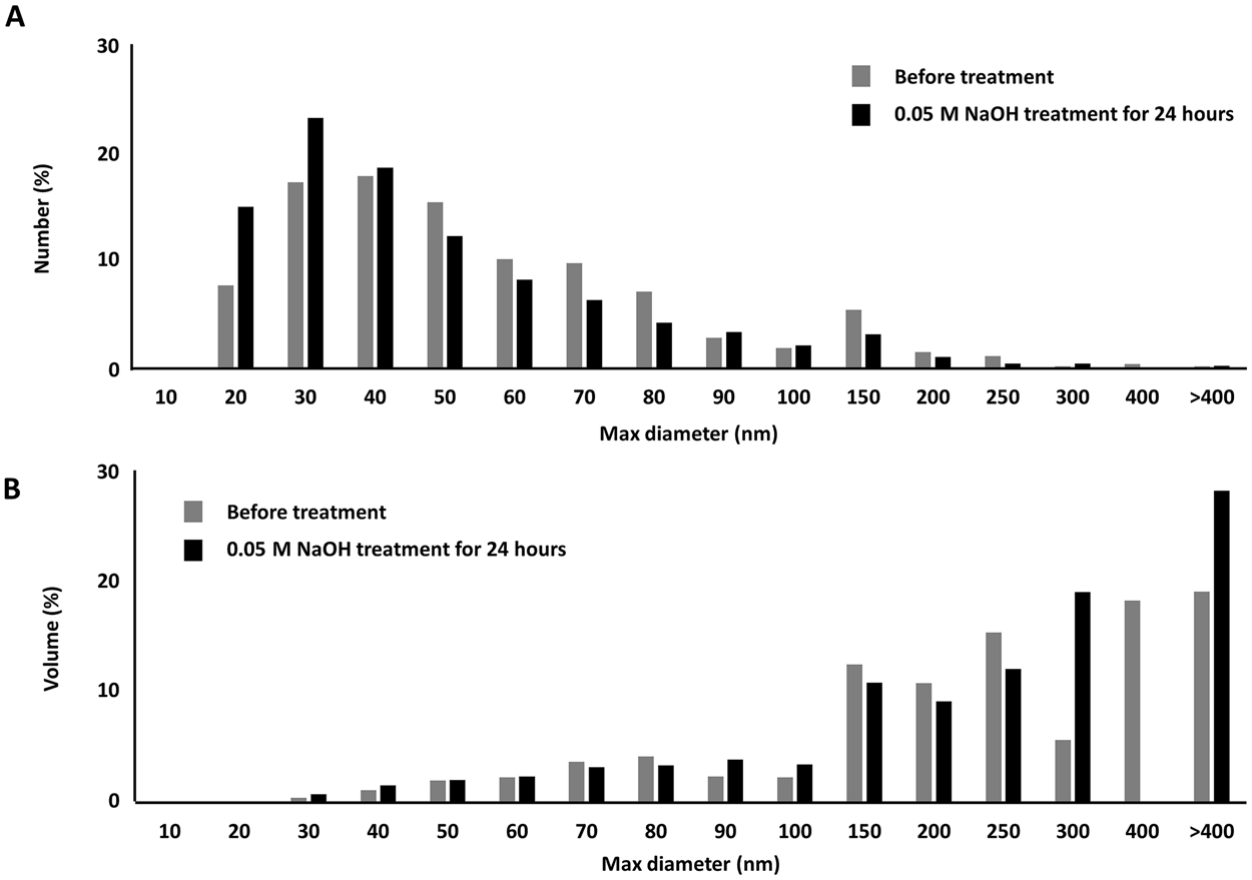
0.05 M NaOH treatment for 24 hours does not alter physical characteristics of MoM Wear Particles. (**A**) Primary particle size for MoM wear particles was measured by TEM before and after 24 hour treatment with 0.05 M NaOH and plotted as distribution by number. (**B**) Primary particle size for MoM wear particles was measured by TEM before and after 24 hour treatment with 0.05 M NaOH and plotted as distribution by volume.

Untreated and 0.05 M NaOH-treated particles were analyzed by TEM for quantitative primary particle size analysis by number and volume after 24 hours of NaOH treatment (Figs 2 and 3). TEM images at the same magnification indicated that 0.05 M NaOH treatment for 24 hours did not alter the physical appearance of the particles (Fig. 2). The mean primary particle size by number before treatment was 55 nm (range: 11–418 nm) (Fig. 3A; Table 2). Similarly, the mean primary particle size by number after 0.05 M NaOH treatment was 46 nm (range: 11–415 nm) (Fig. 3A; Table 2). The mean primary particle size by volume before treatment was 90 nm compared with 82 nm after NaOH treatment; the ranges remained unchanged (Fig. 3B; Table 2).

Morphological differences between particles before and after 24 hours of 0.05 M NaOH treatment were also examined. Length and width information obtained from TEM images were used to determine the aspect ratio of primary particles (Table 2). The majority of particles before treatment were round to oval in shape (62.5 and 35% respectively) and 2.5% were rod shaped with an aspect ratio ≥2.5 (Table 2). Similarly, the majority of particles after 0.05 M NaOH treatment were round to oval in shape (60 and 36.4% respectively) and 3.6% were rod shaped with an aspect ratio ≥2.5 (Table 2). Hence, 24 hours of NaOH treatment did not result in any significant changes in particle morphology.

## Discussion

The main challenge in this study was to develop a method that sufficiently reduced the endotoxin content of MoM wear particles to within the acceptable range of sensitive biological assays while preserving the wear particle characteristics. We initially screened several different sterilization methods including high temperature dry heat, E-beam radiation, ethylene oxide treatment, and a combination of acid/base treatments. The various methods evaluated were either limited by their endotoxin reduction ability or incompatibility with the wear particles, which significantly altered the physical characteristics of the wear particles (Table S1, Figure S1). NaOH treatment was the only method that reduced the endotoxin content of the wear particles and did not alter the wear particles’ physical and chemical characteristics. Thus, we proceeded to optimize the NaOH sterilization method.

Retention of physical and chemical characteristics during sterilization is necessary to preserve the clinical relevance of the particles. For example, physical size and morphology may influence the manner in which the particles interact with biological systems, including particle uptake, clearance, and bioavailability. Similarly, particle chemistry may influence particle interaction with biological tissues, including cellular uptake and processing, tissue penetration and compatibility. The results of this study demonstrated that sterilization by 0.05 M NaOH in 50% ethanol solution can successfully reduce endotoxin content while preserving both physical and chemical characteristics of clinically relevant MoM wear particles isolated from serum-rich lubricant. This study provides the necessary methodology to test clinically relevant particle interactions with biological systems without the confounding influence of endotoxins. Future work can utilize clinically relevant wear particles that are endotoxin-free for various toxicological assays.

Particle yield is crucial as it dictates the amount of depyrogenated material available for further testing. A yield close to 100% would be ideal; however, our study demonstrated an initial decrease in yield that stabilized over time with 92, 78, and 80% yield after 3, 6, and 24 hours of NaOH treatment. The particle yield decreased from 3 to 6 hours, but remained similar between 6 and 24 hours. This trend likely reflects initial dissolution of metal from the particles, followed by a period of stable equilibrium with minimal dissolution of metal into solution. Other mechanisms potentially affecting particle yield include loss due to material storage, transfer, washing or centrifugation. The observed initial loss occurred with similar handling procedures and no change to relative composition and aspect ratio, which suggests a minor non-specific loss of mass due to dissolution prior to establishment of pseudo-equilibrium conditions.

The relative percentage endotoxin reduction increased over time, from 83.354, 98.012, to 99.999% after 3, 6, and 24 hour treatments representing an over 5-log reduction in endotoxin levels after the final time point (Fig. 1). The tradeoff for the endotoxin reduction was a decrease in percentage yield to 92% after 3 hours of treatment which plateaued to 81% after 24 hours of treatment (Fig. 1). Thus, despite the slightly lower yield, the longer treatment was successful at further reducing endotoxin content to a satisfactory level. For example, the 24 hours treatment was associated with an over 5-fold reduction in endotoxin levels approaching 1 EU/mL which is near the FDA endotoxin limit of 0.5 EU/mL for medical device extracts and similar to levels where inflammatory responses are unlikely to occur^18,19,21^.

While the sterilization method developed in this study may successfully reduce the endotoxin content of clinically relevant, serum-based MoM wear particles without altering the physiochemical properties of these particles, further research would be necessary to determine whether the treatment will negatively impact the physiochemical characteristic of other particle types (e.g. titanium or other metal particles). Thus, method screening and further optimization may be necessary for application to other particle types. Additionally, it is possible that several cycles of NaOH treatment may be necessary to obtain acceptable levels of endotoxin depending on several factors including initial contamination levels, particle surface area, and mass. To our knowledge, this study represents the first published sterilization method for clinically relevant MoM hip implant wear particles isolated from serum-rich lubricant. This allows for further application of implant wear particles for various *in vitro* and *in vivo* systems.

## Methods

### Chemicals and reagents

Sterile, endotoxin-free water (#WP0501), control endotoxin standard (#E0005), LAL Reagent (#T0051), Glucashield buffer (#GB051-25) were purchased from Associates of Cape Cod. Ethanol (#111000200) was purchased from Pharmco-Aaper. Nitric acid (#438073), hydrogen peroxide (#H3410), and sodium hydroxide (#S2770) were purchased from Sigma Aldrich.

### Particle generation and isolation

MoM hip simulator wear particles were generated in serum lubricant solution at DePuy Orthopaedics (Warsaw, IN) according to ISO 14242 under previously reported conditions^7^. The simulator was operated at a clinically equivalent angle of 65° and under microseparation conditions. MoM wear particles generated from hip simulator were isolated from serum lubricant at nanoComposix (San Diego, CA) according to previously published procedures and resuspended in sterile H_2_O^7^. Endotoxin content and physiochemical characteristics of isolated wear particles were determined prior to sterilization.

### Endotoxin characterization

Endotoxin content was determined by kinetic turbidity LAL assay at nanoComposix (San Diego, CA) using a Pyros Kinetix Flex 64 Incubating Kinetic Tube Reader (Associates of Cape Cod, MA) according to Nanotechnology Characterization Laboratory method STE–1.2.

### Particle characterization and elemental analysis

Primary particle size of MoM wear debris was determined using transmission electron microscopy (TEM) using a JOEL 1010 Transmission Electron Microscope (JOEL Ltd.). ImageJ image analysis software (U.S. NIH) was used to determine the maximum diameter of each particle. All particles visible in select frames from TEM images were analyzed for length and width. Length of primary particles were measured using the longest chord joining points on the observable perimeter of the particle; width (maximum orthogonal dimension) was also measured. At least 200 particles were analyzed for each sterilization treatment method. Primary particle aspect ratio reflecting morphology was determined from length and width information: particles were classified as round if 1≤ aspect ratio <1.5, oval if 1.5≤ aspect ratio< 2.5, and rod shaped if ≥2.5. Cobalt and chromium metal content of wear debris particles were determined by inductively coupled mass spectrometry (ICP-MS) using a Thermo Fisher X Series 2 (Thermo Fisher Scientific Inc.). The wear particles were digested using a 2:1 ratio of high purity nitric acid (HNO_3_) and hydrogen peroxide (H_2_O_2_) under pressure at 180 °C.

### Optimization of sterilization method

Optimization of the protocol was performed by testing the effects of 0.05 M sodium hydroxide solution (50% ethanol) on endotoxin removal after 3, 6, and 24 hours of treatment. This protocol was optimized and modified from a previous study protocol that reported endotoxin removal for orthopedic particles using treatment with 0.1 M NaOH in 95% ethanol^13^. We performed a combination of tests after each time point including the evaluation of the percentage of endotoxin removed, percent particle yield (total Co and Cr via ICP-MS in treated samples versus untreated), percent Co (of total metal), and percent Cr (in total metal) in the treated samples. Finally, we evaluated the physical characteristics of the treated wear debris particles via TEM analysis.

### Data Availability

The datasets generated during and/or analyzed during the current study are available from the corresponding author on reasonable request.

## Electronic supplementary material

Supplemental Information
