# Supplementary material for: Methods for Sterilizing Clinically Relevant Wear Particles Isolated from Metal-on-Metal Hip Implants
Source: Sci Rep. 2018 Feb 5;8:2384. doi: 10.1038/s41598-017-18239-4 (PMC5799183; doi:10.1038/s41598-017-18239-4)
Supplement: Supplementary file 1 — Supplemental Information [file 41598_2017_18239_MOESM1_ESM.pdf]

# Methods for Sterilizing Clinically Relevant Wear Particles Isolated from Metal-on-Metal Hip Implants

Ernest S. Fung[1], Kenneth M. Unice[2], Dennis J. Paustenbach[3], Brent L. Finley[4], Michael Kovochich[5]*

[1]Cardno ChemRisk; 130 Vantis Suite 170; Aliso Viejo, CA 92656

[2]Cardno ChemRisk; 20 Stanwix St. Suite 505; Pittsburgh, PA 15222

[3]Cardno ChemRisk; 101 2nd St. Suite 700; San Francisco, CA 94105

[4]Cardno ChemRisk; 231 Front Street Suite 212, Brooklyn, NY 11201

[5]Cardno ChemRisk; 30 North LaSalle St Suite 3910, Chicago, IL Illinois 60602

*Please send correspondence to: michael.kovochich@cardno.com

**Keywords:** Cobalt, chromium, nanoparticles, sterilization, depyrogenation, endotoxin, metal-on-metal, wear debris

## Supplemental Information:

**Table S1. Endotoxin reduction and physical characteristics after various sterilization treatments[1]**

| Treatment | % Endotoxin Reduction | Retained Physical Characteristics? |
|---|---|---|
| Dry heat | 99.99 | No |
| E-beam radiation | 67.18 | No |
| Ethylene Oxide | 27.37 | - |
| Acid and Base | 99.97 | No |

[1] MoM wear debris particles were treated with dry heat (up to 300°C for two hours), E-beam radiation (up to 25 kGy), ethylene oxide, or nitric acid followed by sodium hydroxide (acid and base). Endotoxin content was determined by kinetic turbidity LAL assay, particle physical characteristics were determined by TEM.

**Figure S1**

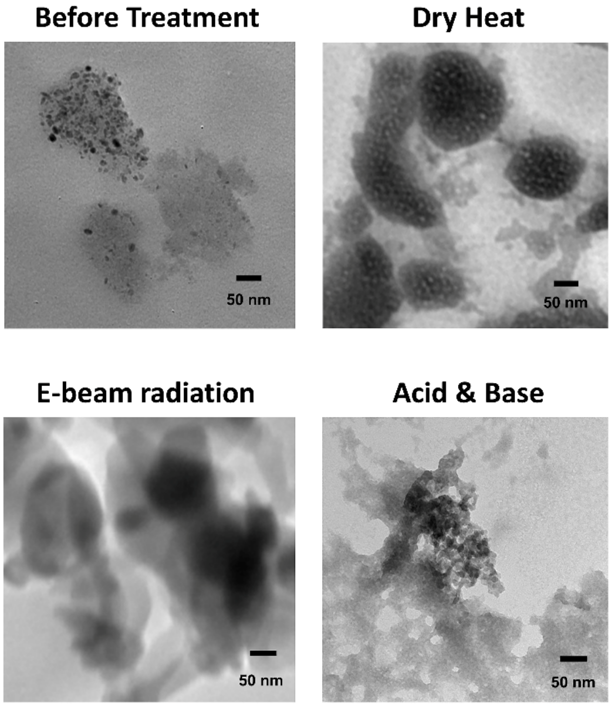


**Figure S1. TEM images of MoM wear debris particles prior to and after various sterilization treatments.** Wear debris particles were treated with dry heat (up to 300°C for two hours), E-beam radiation (up to 25 kGy), or nitric acid followed by sodium hydroxide (acid & base). TEM images were taken to determine the impact of sterilization treatment on physical particle characteristics.
